# Training of psychotherapists in post-conflict regions: A Community case study in the Kurdistan Region of Iraq

**DOI:** 10.3389/fpsyt.2022.947903

**Published:** 2022-08-22

**Authors:** Julia Beckmann, Thomas Wenzel, Martin Hautzinger, Jan Ilhan Kizilhan

**Affiliations:** ^1^Institute for Transcultural Health Science, Baden-Wuerttemberg Cooperative State University, Villingen-Schwenningen, Germany; ^2^Department of Social Psychiatry, University of Vienna, Vienna, Austria; ^3^Department of Psychology, Clinical Psychology and Psychotherapy, University of Tüebingen, Tüebingen, Germany; ^4^Institute for Psychotherapy and Psychotraumatology, University of Duhok, Duhok, Iraq

**Keywords:** war, psychological trauma, psychotherapy, treatment, training, teaching, cognitive behavioral therapy

## Abstract

The number of wars in the world is on the rise. A number of studies have documented the devastating impact on the public and especially public mental health. Health care systems in low- and lower-middle income countries that are frequently already challenged by the existing mental health services gap cannot provide the necessary care for those displaced by war with existing services. This is especially the case in the Kurdistan Region of Iraq (KRI) after the invasion of the terror organization ISIS in 2014. Most projects in post-conflict areas focus on short term basic psychological services and do not contribute to sustainable long-term capacity building of mental health services. An “Institute for Psychotherapy and Psychotraumatology” was therefore founded in order to train local specialists on a professional level with evidence-based methods adapted to culture and create sustainable long-term structures for psychotherapeutic treatment in the KRI. To achieve this, a number of measures were implemented, including the creation of a “Master of Advanced Studies of Psychotherapy and Psychotraumatology” in collaboration with local communities and the regional University. Two cohorts of students have successfully finished the master’s program and a third cohort are expected to graduate in 2023. Improving the capacity of local health care services to provide low-barrier, professional psychotherapeutic care in post-conflict regions supported by the innovative model presented in this article can be expected to improve the burden of psychological problems and contribute to peacebuilding.

## Introduction

At the end of 2020, 82.4 million people worldwide were fleeing persecution, conflict, violence and human rights abuses according to UNHCR. This number has doubled in comparison to 10 years ago (1). The number of wars and conflicts in the world has increased again since 2013 (2). Many of the active conflicts are long-standing or resurgent and most conflicts occur in low- or lower-middle-income countries (3, 4). About 86% of those fleeing live in poor countries (1) with very limited health care, and especially limited mental health services.

Due to modern warfare, increasing numbers of those who suffer in conflicts are civilians (3, 5, 6). It has been previously described that most people in conflicts experience at least one traumatic event in the form of extreme violence, terrorist attacks, kidnapping, or torture (7). Flight, displacement, and loss of loved ones tear apart families and communities, which usually serve as important social support networks (8). Furthermore, precarious living conditions after flight due to destroyed infrastructure, lack of access to social services and living in refugee camps (for often long term periods with no perspective) contribute to exacerbating the situation (6, 9–11).

About 50% of severely traumatized people suffer from trauma induced disorders, of which 25% become chronic (12). This can lead to long-term individual suffering and transgenerational trauma (13), as well as negative long-term consequences for society, such as loss of labor force or financial cost (14). The most frequent diagnoses in post-conflict regions are posttraumatic stress disorder (PTSD; 3–88%), Major Depression (MD; 5–80%) and anxiety disorders [1–81%; (15, 16)].

### The psychological impact of war and flight using the Kurdistan Region of Iraq as an example

Iraq has a population of about 40 million inhabitants, most of whom are Muslim Arabs. The second largest population group are Kurds. In addition, there is a large number of ethno-religious minorities (17). For decades, the country has been in wars or warlike conditions. In northern Iraq lies the Kurdistan Region of Iraq (KRI), which established its *de facto* autonomy from Iraq in 1991. In 2005, the Baghdad government recognized the autonomy of the region officially in its constitution. About 5 million people live in the KRI.

Iraq has been particularly affected by recurrent war and conflict in recent decades: the Iran-Iraq War (1980–1988), the Gulf War (1990–1991) and the Iraq War after the United States invasion (2003–2011). The Iraq Mental Health Survey 2006/7 (18) is the first and only extensive survey of mental illnesses in Iraq. The survey states 12-months-prevalences of 3.99 for any affective disorder, 1.63 for PTSD and 8.58 for any anxiety disorder (including PTSD). More recent research on mental illness in Iraq focuses on specific settings. According to Freh (19) for example, one decade after the US invasion in 2003, prevalences measured in the year 2013 were 55.8% for PTSD and 63.4% for MD among young adults affected by political violence.

In 2014, the so-called “Islamic State” (ISIS), a terror organization, invaded Iraq and captured provinces in the west and north of the country. Seen as “infidels,” religious minorities, particularly the Yazidi people, were treated with extreme brutality. Men were executed, women and children abducted and raped, boys turned into child soldiers (20). This led to massive refugee movements in northern Iraq and Syria.

Due to the extreme brutality, the continuous danger and the long-term displacement of large groups of the population within the country, psychological problems and mental illness are particularly common here. Approximately 79% of Yazidi internally displaced persons (IDPs) in a refugee camp in the KRI reported PTSD symptoms (21). Among Syrian refugees in northern Iraq, a prevalence of 35–38% for PTSD related to trauma and torture was found by Ibrahim and Hassan (7). These numbers coincide with more recent findings about IDPs in Dohuk province by Taha and Sijbrandij (22), who report a rate of 29.1% for PTSD among female IDPs and 31.9% among male IDPs. The rate of common mental disorder cases was 65.1% among female IDPs compared with male IDPs (55.4%). Prevalences are especially high among vulnerable groups that experienced especially severe traumatization: In former child soldiers that had been recruited by ISIS, prevalences of 48.3% for PTSD, 45.5% for MD and 45.8% for anxiety disorders have been previously reported (23). Among Yazidi women who had been victims of the 2014 genocide and had been in captivity of ISIS for months, Kizilhan et al. (24) found prevalences of 97.9% for PTSD and 88.1% for MD.

In addition to the influence of traumatic experiences during conflicts, the local conflict aftermath seems to play a significant role in the development of mental disorders, particularly increasing the risk for MD (9, 25, 26). Refugee and IDP camps in the KRI are slowly becoming a permanent shelter for many people since 2014. As of September 2021, about 1.2 million internally displaced persons (IDPs) and 248,000 Syrian refugees remain in Iraq, the majority of whom are in the KRI (27). In August 2021, there were still about 183,000 people living in 27 refugee camps in all of Iraq, most of them in the KRI [only two of the 27 camps are in central Iraq; (27)]. There is also a large number of IDPs living in informal settings outside of the camps. For many of them, returning to their homes is not an option because of destruction and ongoing safety concerns.

Life in the refugee camps is characterized by extremely poor living conditions. They do not offer sufficient infrastructure and health services to address the multiple needs, in spite of the activity of numerous non-governmental organizations (NGOs) active in the region (28). Mental illness is a common reason for seeking treatment, particularly among adult women in camps (29). While care services are scarce in camps, many affected people seek help outside. However, in addition to the cost for treatment and transportation, the stigma of mental illness is a major barrier (28). Finally, yet importantly, after decades of conflict without an adequate psychosocial care system, a high number of mental illnesses can also be assumed in the host community.

### Psychosocial care in post-conflict regions using the Kurdistan Region of Iraq as an example

Kizilhan (30) describes the necessary steps of care after disasters like war and conflicts: Before psychosocial interventions are implemented, it is crucial to bring people to safety, care for basic physical needs and provide medical first aid (see Table 1). While this is done, the principles of psychological first aid come into play, which encompass the protection from further harm, care for basic needs, the opportunity to talk about the events and guidance toward helpful coping strategies (31). While these steps are relevant for most of the affected people in a crisis region, only part of them will develop a stress or trauma related mental health problem or aggravation of a preexisting condition. The development of further mental illness depends on several pre-, peri- and post-traumatic risk factors (e.g., the kind of trauma experienced). As described above, because of the severe brutality used by ISIS, the number of people affected by lasting mental illnesses is high in the KRI. For people who develop trauma related sequelae, psychological examination and treatment by trained mental health care experts are necessary.

**TABLE 1:** Interventions after disasters [adapted from Kizilhan (30)].

Intervention	Target population	Examples of Interventions	Intervention conducted by
Finding people and protecting them from further threats	Most of the people who are affected	Search for missing persons, find shelter	Military, police, community
Take to a safe place if possible	Most of the people who are affected	Put in a safe place (house, tent, etc.)	Police, health staff
First medical emergency aid	Most of the people who are affected	Physical examination, first treatment of injuries	Physicians, aid workers
Medical examination	Most of the people who are affected	Physical examination, outpatient or inpatient treatment	Physicians
Psychological first aid	Most of the people who are affected	Restoring immediate safety, restoring contact with loved ones	All responders, aid workers
Psychological examination and diagnostics	People with first psychological symptoms, like acute stress disorder	Address anxiety, state of shock, first depressive symptoms, sleeping disorders, etc.	Psychiatrists psychotherapists, clinical psychologists
Psychological examination and diagnostics after one month	People who meet the criteria for PTSD	Psychological screening and assessment, long- term psychotherapy	Psychiatrists psychotherapists, clinical psychologists
Skills for psychosocial recovery	People whose distress is sustained by bereavement or secondary stressors	Assessment of needs, problem-solving, social support	Healthcare practitioners, workers with expertise in the required skills and in conveying these skills effectively
Psychosocial interventions for medium- and long-term problems	People whose distress is sustained and associated with functional impairment	Culture sensitive adapted psychotrauma-therapy, outpatient and/or inpatient psychiatric treatment	Staff of mental healthcare facilities, psychiatrists psychotherapists, clinical psychologists, social workers specialized in trauma

However, 76–85% of people with severe mental disorders in low- and middle- income countries do not receive treatment (14). Almost half of the population worldwide lives in countries where, on average, one psychiatrist serves 200,000 or more people. Other health care personnel trained for treating mental disorders is even rarer (14).

In order to close the gap between high demand and little specialized care, many poor countries apply the concept of task shifting: Mental health care is integrated into primary health care by training general practitioners and other non-specialized health care personnel like nurses or social workers (32). Task shifting falls under the Mental Health and Social Support (MHPSS) approach, which comprises any external or local support provided to protect or improve mental well-being (31). This includes interventions provided by specialized healthcare workers as well as techniques implemented by trained laypersons. International NGOs, which often support the few local care structures, frequently use this principle.

Many of the temporary symptoms and discomforts that occur after a disaster can be addressed through MHPSS approaches. Task-shifting has also been used in Iraq and proves effective in reducing psychological symptomatology (32). However, the development of PTSD or other severe and long-lasting mental disorders cannot be prevented by these approaches (30). The treatment of these disorders should be carried out by specialized personnel such as psychiatrists or psychotherapists, which, as reported, is hardly available in Iraq (see Table 2). According to the Iraq Mental Health Survey 2006/7 (18), in the Kurdistan region there were 17 general psychiatrists, 2 psychiatric practitioners, 4 child and adolescent psychiatrists, 91 psychiatric nurses, 4 psychologist, 15 social workers and 2 psychotherapists at that time. Economical and geographical barriers, such as the lack of public transport and high cost of medication and services, add to the dire situation. This leads to limited *de facto* access especially for groups most in need.

**TABLE 2:** Details on personnel working in mental health (per 100,000 inhabitants) in Iraq and its neighboring countries.

Country (Year of data collection)	Psychiatrists	Nurses	Social workers	Psychologists
Iraq (2017)	0,343	1,218	0,089	0,111
Iran (2017)	2,016	9,451	1,512	5,166
Jordan (2016)	1,125	3,297	0,218	1,266
Saudi Arabia (2016)	1,321	10,660	3,955	2,034
Syria (2016)	0,368	1,068	0,801	1,068
Turkey (2016)	1,637	150,251	1,643	2,537
United States (2016)	10,542	4,283	60,338	29,864
Germany (2015)	13,202	n.a.	n.a.	49,555

Data from Germany and the United States provided for comparison, according to the (49).

In the KRI more humanitarian assistance is available for refugees and displaced persons due to the relatively stable security situation in comparison to central Iraq (33). However, due to the better security and the familiar environment and language, significantly more displaced people from Syria and all of Iraq also come to the region. The number of severely traumatized, psychologically burdened people is therefore high, and so the demand for psychological aid is immense. This leads to an overload of any of the existing local services already taxed by low capacity before 2014 and by a consistent brain drain of trained experts (34). NGOs located on site are able to address some of the need with the aforementioned MHPSS interventions. However, the high demand often means long working hours and low quality of work for the few professionals. There is a risk that NGOs will employ under-qualified practitioners for the treatment of severe mental diseases such as PTSD (35). Additionally, the difficult working conditions pose a risk for secondary traumatization (36). International NGOs usually stay on site for a limited amount of time: While there were 24 international NGOs in Iraq in 2016 (37), there were only 11 remaining in July 2021 (38).

### Rationale for improving psychosocial care in the Kurdistan Region of Iraq

Due to the development described above, the need for sustainable local services and trained local experts becomes even more pressing, especially considering the risk of long-term suffering and transgenerational transmission of untreated trauma.

The Iraqi health care system does not provide the necessary care. In the aftermath of long-lasting conflicts, economic difficulties and destruction of the necessary infrastructure, the system deteriorated increasingly since the late 1980s (39). In the area of mental health, there is a significant lack of specialized professionals. This is partly because there is no specialization in clinical psychology or psychotherapy, neither in health care training nor at universities or medical schools. No distinction is made between psychotherapists and other health care professions such as social workers or counselors (40). No law exists regulating psychotherapy and “psychotherapist” as a profession is largely unknown.

The situation described prompts the formulation of two objectives for the proposed innovation presented here: First, the improvement of low-barrier psychotherapeutic care. Second, the creation of academic structures that ensure the permanence of the training of professionals in the region and contribute to a sustainable solution.

## Description of the proposed innovation: Sustainable training of local psychotherapists

Aiming to train local specialists in the KRI and create sustainable structures for psychotherapeutic treatment, the “Institute for Psychotherapy and Psychotraumatology” (IPP) was founded in 2016 with the help of the state government of Baden-Wuerttemberg under the coordination of Prof. Dr. J.I. Kizilhan and Prof. Dr. M. Hautzinger. Subsequently, the “Master of Advanced Studies of Psychotherapy and Psychotraumatology” (MASPP) was established.

A number of key factors guided the development and implementation of the IPP and MASPP: (a) the training should be grounded in evidence based international standards, (b) training should be culture and trauma sensitive, (c) a core group of local professionals should be trained that can provide training of further local therapists, (d) the project should be coordinated with local leaders, the local government, health care services, and academic institutions, (e) a long term collaboration between the local group and academic institutions with international partners should be built, (f) research should be encouraged to provide more information on needs and project outcomes, (g) a free, low barrier model outpatient service should be implemented, (h) outreach activities should be included to address mental health stigma. Examples of the measures taken to include these considerations are listed in Table 3.

**TABLE 3:** Considerations and key factors that guided the development of the institute for psychotherapy and psychotraumatology with examples for their implementation.

Guiding key factors and considerations	Examples for measures taken
Evidence based international standards	Focus on cognitive behavioral therapy and narrative (trauma) therapy, with leading international experts as trainers in the pilot project
Culture sensitivity	Including all local ethnic and religious groups in planning and training, supporting a focus on transcultural adaptation of international standards
Building a core group of therapists to provide further training	“Train-the-trainer” program, training graduates of the first cohorts to become lecturers
Coordination with local stakeholders	Close collaboration with local government, religious community and minority leaders and NGOs, embedding the IPP in the structures of the University of Dohuk
Integration in international professional and academic networks	Stable collaboration with international universities [in Germany especially the University of Tuebingen and the Baden-Wuerttemberg Cooperative State University (DHBW)], and professional umbrella organizations (such as World Psychiatric Association), joint international conferences in Duhok
Integration of research strategy	Teaching scientific methods as part of the master’s program, encouraging post gradual research, regular research projects published in international and local journals
Implementation of a free, low barrier model outpatient service	Implementation of the *German Clinic for Psychotherapy*, embedded in the public health service center with local and international staff, with a suicide prevention center as most recent service
Outreach addressing stigma	Social media and other media created by students, in addition to regular collaboration with public media, government and NGOs regarding the stigma challenge, inclusion of trusted community members and leaders as a bridge to the public

Since IPPs opening, two cohorts have completed the master’s program successfully, with a third cohort completing the program in 2023. In the meantime, a part of the previous graduates has been further trained through a “train-the-trainer” approach, in order to assume responsibility for teaching and other institute activities in the future and thus ensure sustainability. The IPP and the master’s program are supported and funded by the Ministry of Science, Research and the Arts of Baden-Wuerttemberg, the German Foreign Ministry and the German Academic Exchange Service. Details on each aspect of the project are presented below:

### Institute for Psychotherapy and Psychotraumatology

The IPP aims to research the basic principles and application of psychotherapy and psychotraumatology and to further develop culturally sensitive approaches for use in therapy. Additionally, the institute supports the students of the master’s program to obtain the dual degree of master’s and license to practice as psychotherapists, and to make use of the opportunity to pursue doctoral studies. There is close cooperation of IPP with the University of Dohuk, the Ministry of Higher Education and the Dohuk Health Directorate.

### Master of Advanced Studies of Psychotherapy and Psychotraumatology

The master’s program teaches the theoretical principles and the practical implementation of psychotherapy and psychotraumatology. Upon graduation, students can work as highly qualified professionals in health care institutions. A prerequisite for participation is the completion of a bachelor’s degree in psychology, social work or education. Applicants have to participate in admission interviews regarding their professional and personal competence. As the main teaching language is English throughout the whole program, participants have to show proof of sufficient language skills.

The curriculum developed for the master’s program is based on the German examination regulations for “psychological psychotherapists” (i.e., licensed psychologists that after successfully completing a diploma or master’s degree in psychology have completed at least 3 years of full-time training) and thus offers all teaching contents of psychotherapist training according to German standards. However, due to the local situation and the high number of traumatized men and women in the KRI, emphasis was placed on teaching techniques for the treatment of trauma sequelae and on teaching transcultural aspects of therapy. The parallel integration of theoretical, practical and self-reflective modules under supervision enables a deep understanding of psychotherapy and therapeutic impact factors.

Contents of the curriculum are: (a) basic knowledge (theories, models) of psychology, psychotherapy and psychotraumatology, (b) assessment, functional analysis of problems, classification and diagnosis of mental illnesses, (c) methods of scientific work and evaluation, (d) clinical skills, treatments and intervention methods, (e) cross-cultural issues of therapy. The fundamentals of all mental disorders and their treatment are taught and applied practically in small groups and role-plays.

**FIGURE 1:**
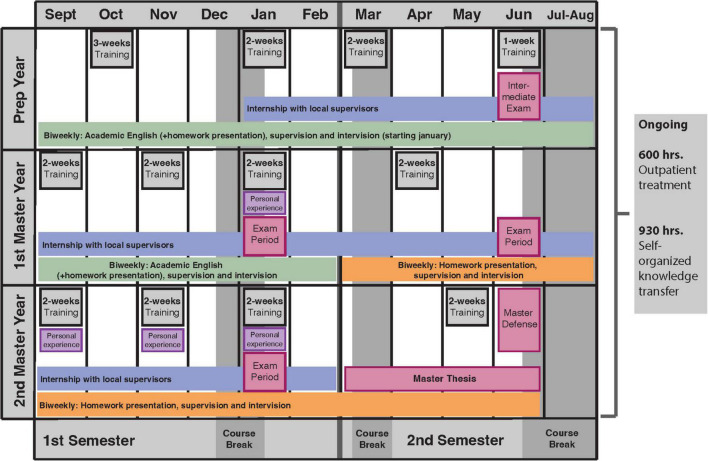
Course program for the 1st and 2nd semester.

The program consists of a preparatory year and a 2-year master’s program, which concludes with a master’s thesis and defense. The detailed sequence of the components over the course of the program is shown in Figure 1. The individual elements are:

1. Theoretical training (600 units of 45 min).
2. Practical experience in psychiatric institutions, refugee camps, clinics (1,800 units of 60 min).
3. Treatment of outpatients with various mental disorders (600 units of 50 min).
4. Clinical supervision (150 units of 50 min).
5. Personal experience, which encompasses reflecting one’s own person and experiencing therapy methods in the client role (120 units of 50 min).
6. Independent knowledge acquisition by means of eLearning modules (120 units of 45 min), homework (370 units of 45 min) and treatment documentation (440 units of 45 min).

Because English is the main teaching language, the preparatory year includes English classes. At the end of the preparation year, students must pass an exam regarding their language skills in order to continue the program.

The teaching is supported by eLearning content on the ‘‘moodle’’ platform.^1^ Both, the Baden-Wuerttemberg Cooperative State University and the University of Dohuk were already using “moodle” as an eLearning platform beforehand. The content was developed specifically for the project in collaboration between the local and international staff. This ensures continuity of cooperation in the context of international collaboration in phases of limited security, as well as in the context of the current COVID-19 pandemic. Access has been provided to international professional online journals and books through the library of the University of Tuebingen.

### Sustainability

The first cohort starting in 2017 was taught mainly by international lecturers and experts. To ensure sustainability, a “train-the-trainer” approach was planned from the outset, with some of the graduates of the first cohorts being trained as lecturers and increasingly assuming responsibility for teaching and administrative activities at the institute. The students selected for this purpose take part in additional training courses on study organization and management as well as on didactics. They support the external experts as co-lecturers in order to gain first practical experience in teaching and supervision, before taking over teaching under supervision by an international lecturer. It is planned that the University of Dohuk assumes full responsibility for institute activities and funding in the long term.

The long-term implementation of nationwide psychotherapy can only succeed if the profession of licensed psychotherapist receives recognition and is distinguished from other professions. Therefore, efforts are made to integrate the profession of psychotherapist legally into the Iraqi health care system. There are also efforts to stimulate and strengthen the cooperation between psychiatrists and psychotherapists.

Finally, yet importantly, awareness about traumatizing events and mental illnesses must be raised among the population in order to improve psychotherapeutic care sustainably. In Iraq, as in the entire Middle East, there is great stigma regarding mental illness (28, 35). IPP’s activities therefore include networking with local institutions and NGOs working in the health sector to stimulate public discussion among the population, create acceptance, and lower the threshold for seeking psychotherapy. IPP‘s students and graduates as well as their patients also become multipliers who spread knowledge about psychotherapy in society and contribute to reducing stigma.

## Initial measurable results and successes

In April 2020, 28 of the original 30 students successfully completed the master’s program. Out of the second cohort, which started in October 2018 with 24 students, 21 participants graduated in November 2021. The third cohort of 25 students began the preparatory year in October 2020 and will complete the master’s program in fall 2023. After graduation, most psychotherapists work in high positions in local or international NGOs.

Five students out of the first cohort and two students out of the second cohort were trained as co-lecturers in the “train-the-trainer” program to assist with teaching, supervision, and personal experience. Some of the co-lecturers are pursuing doctoral degrees at the same time.

The 30 students in the first cohort treated 842 patients in 11,563 therapy sessions as part of the practical parts of their master‘s studies between 2017 and 2019. The students of the second cohort treated 891 patients in 12,452 sessions between 2018 and 2021. Hence, in average 13.9 therapy sessions per patient were conducted.

In February 2021, the “German Clinic for Psychotherapy,” an outpatient clinic belonging to the IPP was established. There are currently eight graduates of the IPP working there as psychotherapists, treating people from the refugee camps as well as from the city. The treatment is free of cost for the patients, as high fees constitute a barrier to seeking help beside stigma. A local community member with similar linguistic and ethnic (Yazidi) background from the community has been installed as a first contact to explain services and answer questions about psychotherapy, further reducing fears and stigma.

In addition to teaching and training psychotherapists, IPP successfully organized two international conferences. A conference in 2018 focused on genocide and trauma, and in 2019, a conference focused on the adaptation of Western and Middle Eastern therapeutic methods. Both conferences were well attended, bringing together local and international experts and stakeholders.

## Discussion

In this work, we describe the first establishment of a postgraduate psychotherapy training program in the KRI. The goal of the intervention described was to implement a sustainable solution to the inadequate mental health care in the KRI by having psychotherapeutic care provided by local professionals and integrated into the health care system. We are hoping for this pilot project to serve as best practice example for other post-conflict regions to help set up sustainable psychosocial care in other parts of the world.

The establishment of the IPP and implementation of the master’s program demonstrate that the training of psychotherapists and the integration of psychotherapeutic care into the health care system have started successfully. Many of the lessons learned in the process echo those from WHO’s Building Back Better report (41) which uses 10 countries as examples to demonstrate how crises can act as a catalyst for a positive, sustainable development of health systems. In particular, it shows the importance of successful long-term planning from the outset and of incorporating the central role of government. Close cooperation with the Ministry of Higher Education and the Health Directorate played a central role in facilitating the integration of psychotherapy training into the education system and the inclusion of psychotherapy in the health care system. A next step is to embed the profession of psychotherapist legally, in order to give local professionals the recognition they deserve (including financial recognition) and to make psychotherapy more easily distinguishable from other health professions.

In terms of teaching content, a successful transfer of German psychotherapy training to another culture is shown, with some necessary adjustments to the curriculum. Due to the high number of traumatized people in the KRI, the curriculum focuses on trauma-specific treatment approaches. Those are addressed already very early in the program. However, as required by the German psychotherapy training, students are trained to treat all mental illnesses. This is in contrast to existing MHPSS approaches like the “mental health Gap Action Program” (mhGAP) or the “Problem Management plus” (PM+) by the WHO, which focus on teaching “low intensity psychological interventions” to non-specialized care providers (42, 43). The broad teaching contents, especially the inclusion of clinical supervision and personal experience, in our master’s program make the graduates of IPP capable of diagnosing and treating all kinds of mental illnesses and reflect on their interaction with the patient and the therapy process as a whole. They are able to adapt therapeutic methods to new contexts and understand the possibilities and limits of psychotherapy. This enables them to work in many different contexts and with different target groups and, in the long term, also to treat mental illnesses that are independent of conflict, hence ensuring sustainable mental health care in the region for all kinds of problems.

The curriculum also focuses on addressing cross-cultural differences and teaching culturally sensitive treatment approaches. Religious and cultural beliefs influence the perception and interpretation of psychological symptomatology and therefore must be considered in treatment (44). In particular, potential interreligious conflicts, such as those found in the KRI, should be considered: As a result of the brutality used by ISIS against those of other faiths, patients who are Yazidi, for example, may express mistrust of Muslim therapists (45). Moreover, in traditional, collectivist societies, relationships within the family and the well-being of the entire social fabric play a greater role than individual needs. Therapeutic approaches oriented to Western values, which mainly focus on the individual well-being of the client therefore need to be adapted. Furthermore, cultural differences need to be taken into account regarding the use of didactic methods in the training of students. For example, because of a much more hierarchical instruction style in Iraqi teaching, students are not accustomed to class discussions where they must stand out and apply critical thinking (46). Cultural differences must also be reflected upon with students in the context of culturally sensitive clinical supervision and personal experience sessions (45).

Next to teaching, another goal of IPP is to research culturally sensitive treatment methods and to develop corresponding materials. In addition to the Trauma Workbook (47) which supports students learning about trauma therapy, a culturally sensitive personal experience manual is currently being developed. The cooperation between international lecturers and their corresponding local lecturers that were part of the “train-the-trainer” program has inspired scientific cooperation and common work on transcultural research questions. For all parties involved, the described intervention also contributes to personal growth and cross-cultural understanding and openness.

The success of the IPP is evident in the high acceptance of therapy by patients, the distinctly high demand for graduates of the MASPP as employees from NGOs and other institutions, and the continuing support by the University of Dohuk. In order to stabilize the operation, in addition to a comprehensive ongoing evaluation of the steps taken, the handover of all institute activities to the University of Dohuk, the expansion of the institute’s outpatient clinic and the establishment of an additional outpatient clinic are planned.

Last but not least, the project will hopefully contribute to increasing awareness of mental illness and its consequences and thus increase the acceptance of psychotherapy in the region. At the same time, the project serves as a model that can be emulated and adapted to other world regions affected by violence. The consequences of violent conflicts (PTSD, depression, feelings of anger and revenge, and many more) can make social coexistence difficult and hinder a peace-building process (48). Psychotherapy can help to cope with these feelings in a productive way, thus representing an important contribution to the reduction of war related suffering, which, if it remains untreated, can lead to transgenerational transmission of trauma. Psychotherapy therefore poses an important contribution to peacebuilding.

## Data availability statement

The original contributions presented in the study are included in the article/supplementary material, further inquiries can be directed to the corresponding author.

## Author contributions

JB wrote the main article. TW, MH, and JK read and approved the final manuscript. JK as head of Institute gave detailed information and background about the current situation in Iraq and the Master Program. All authors contributed to the article and approved the submitted version.

## Footnotes

1
https://moodle.de/
